# Supplementary material for: Effect of Combining Low Temperature Plasma, Negative Pressure Wound Therapy, and Bone Marrow Mesenchymal Stem Cells on an Acute Skin Wound Healing Mouse Model
Source: Int J Mol Sci. 2020 May 23;21(10):3675. doi: 10.3390/ijms21103675 (PMC7279345; doi:10.3390/ijms21103675)
Supplement: Supplementary file 1 [file ijms-21-03675-s001.pdf]

Mouse pressure ulcer models

Male ICR mouse (6 ~ 8 weeks) were purchased from Koatech Laboratory Animals, Inc. (Pyeongtaek, Gyeonggi, Korea). All animal experiments were followed by compliance with the Guide for Care and Use of Laboratory Animals of the National Institutes of Health, and the protocol approved by the Animal Research Ethics Board of Hallym University (HMC2017-2-1121-30). The mice were anesthetized, and maintained using 100 % oxygen with 2.5% isoflurane (Hana Pharm, Seoul, Korea). Back of mice was shaved and sterilized with 70% ethanol. The back skin was gently pulled up and sandwiched between two round type ceramic magnetic plates that were 12 mm in diameter and 2.0 mm thick, with an average weight of 1.2 g and 1200 Gauss magnetic force, which generated a 5.0 mm skin thick bridge between the two magnets on four mice per experiment (Fig. 4A). Placed the magnet for 12 hours and the remove time was 12 hours as single ischemia-reperfusion cycle (I/R) cycle. During that time, mice allowed food and water ad libitum. After three I/R cycles, the wound was treated with LTP for 3min a day for 7 consecutive days. The wounds allowed to expose to the air and to natural drying forms scab. Mouse were sacrificed on day 0, 3, 5 and day 7 after wounding. The area of 12 mm in diameter was obtained and used for western blot analysis.

Results

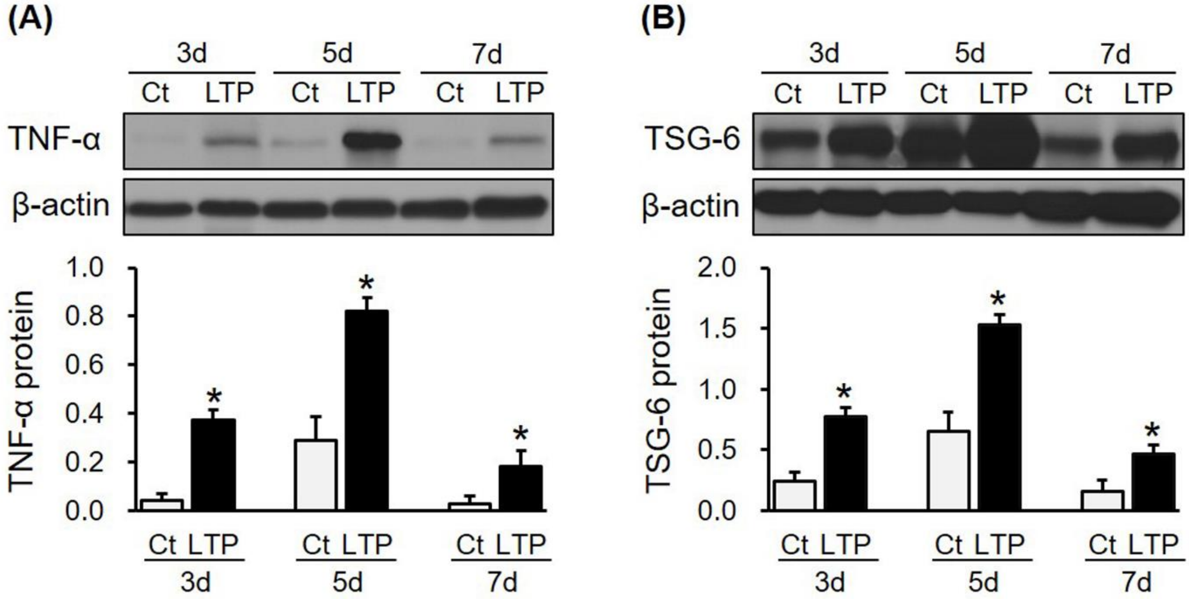

Supplementary Figure 1. LTP treatment induced expression of TNFα (A) and TSG-6 (B) on wound tissues in excisional acute skin wound model of mouse. Wounds were treated with LTP for 3min per day and that samples obtained at 3, 5, 7 days, respectively. Protein expression levels are normalized with β-actin. Data are the mean S.E. (n=4), *$p < 0.05$ vs. that of the control group. Ct, un-treated controls; LTP, Low temperature plasma.
